# Categorizing short term readmissions in an academic emergency department in North India: exploring approaches to reduce them

**DOI:** 10.1186/1865-1380-8-S1-O2

**Published:** 2015-04-22

**Authors:** Dolly Chandrabhan Yadav, Prasad Chidanandan Siddalingeswara, Tamorish Kole

**Affiliations:** 1 Max Superspecialty Hospital, New Delhi, India

## Objective

Categorizing short term readmissions in Emergency Department (ED) and exploring methods to reduce avoidable ED readmissions.

## Introduction

Readmissions in the ED are multifaceted. A recent meta-analysis showed that less than a quarter of readmissions could be considered avoidable [1]. Less is known regarding the systemic causes of readmissions [1]. Research till now, has focused on the risk factors predictive of readmissions and identifying the most common diagnosis [2-4]. A study was undertaken to identify factors associated with this quality care indicator.

## Method

Prospective observational data from electronic hospital and patient health records were collected from 1^st^ September 2013 through 31^st^ August 2014 at Max Superspecialty Hospital, Saket, New Delhi, India. Number of readmissions within 72 hours of index ED registrations were considered. Exclusive and hierarchical categorizations of readmissions within 72 hours of index ED registrations were done under the following categories: (1) Avoidable readmissions- (1a) Readmissions due to inadequate care, (1b) Readmissions due to poorly managed transitions during discharge; (2) Unavoidable readmissions- (2a) Readmissions due to complications, (2b) Readmissions due to recurrences; (3) Unrelated readmissions (different body systems); (4) Other planned readmissions; (5) Readmissions after LAMA (Leaving Against Medical Advice). Statistical analysis was done using SPSS 16.0 and cross-tabulation technique was applied on patient variables.

## Results

A total of 19,205 ED registrations took place from 1^st^ September 2013 through 31^st^ August 2014. Of these 473 patients (2.46%) were readmitted within 72 hours of their index ED registrations. Rate of short term ED readmission and ED registrations showed minimal monthly variability. The mean age of patients was 43 years. Amongst the short term ED readmissions, males (253) outnumbered females (220) marginally. Of 473 short term readmissions, 181 (38%) were avoidable readmissions followed by 86 (18%) unrelated readmissions followed by 82 (18%) unavoidable readmissions followed by 76 (16%) who were readmitted after signing LAMA followed by 48 (10%) planned readmissions. In the avoidable short term ED readmission category, 66 (36%) readmissions were due to inadequate care while 115 (64%) were poorly managed transitions during discharge from the ED. In the unavoidable short term ED readmission category, 44 (52%) patients got readmitted due to complications while 38 (48%) were due to recurrences of signs and symptoms.

## Discussion

A readmission could be due to healthcare factors- hospital or primary care, or patient factors- disease and management plan understanding, compliance and adequate follow up. Our analysis suggests that improving the transition during discharges by bridging the gap and sustaining the quality of care between hospital and home can improve patient outcome.

For this, interventions need to be targeted at three levels [5]: (1) Pre-discharge interventions- discharge planning including patient education (2) Post-discharge interventions- home care and ambulance services, appointing case managers or transition care managers (3) Administrative interventions- ED readmission policy, readmission prevention checklist and automated indicators in electronic hospital and patient health records.

## Conclusion

There needs to be further studies and analysis identifying causes of readmissions and ways to reduce them.
